# Comparative Anatomy of the Nervous System

**Published:** 1840-10

**Authors:** 


### ART. XII.

*Illustrations of the Comparative Anatomy of the Nervous System.* By JOSEPH SWAN.—*London*, 1839. Part V. 4to, pp. 107 to 195.

IN noticing this fifth number of Mr. Swan's "Illustrations," we cannot do less than repeat the very favorable opinion we have before given of the general character of the work, as that of a most skilful anatomist. When completed it will justly be entitled to a place in every library, beside that of its great predecessor by the same author, The Nerves of the Human Body.

The present number contains only two plates; but these are in illustration of that part of anatomy in the higher mammalia upon which Mr. Swan's great excellence as an anatomist of the nervous system has been established—the nerves of the viscera. We have on the whole derived more satisfaction from the perusal of this than of the preceding numbers, since it contains a comprehensive description of the vagus and sympathetic nerves in a large number of mammalia, with details of the peculiarities of these in different species, all of which are highly valuable, and will assist to rectify many of the contradictory statements of physiologists, who have experimented on animals with a view to determine the functions of these nerves, and their peculiar influences on different organs. From the details now given by Mr. Swan, it will be seen that there are good reasons for believing that many of the contradictory statements above alluded to, have arisen from the experimenters having often mistaken the nerves upon which they have operated; or perhaps have ignorantly included others in their trials; or even, perhaps, have entirely missed their object, through want of correct knowledge of the anatomy of the parts implicated in their experiments. We have ourselves witnessed instances of some of these things having occurred even in the performances of those who have gained some notoriety for experimenting, and have heard, with much amusement, the ingenious explanations which have been given to such experiments, when the results have differed greatly from those previously predicted by the operator. We have not the slightest doubt that instances of this kind have frequently occurred, and have been the source of much error in the statements of those who are desirous of obtaining a reputation for experimenting, without sufficient preliminary knowledge to render them fitted for so responsible a task. We cannot too strongly condemn such a mode of proceeding; and we would therefore strongly recommend to those who are ambitious of becoming, not mere experimenters, but promulgators of truth, first of all to fit themselves by carefully studying, not generally but minutely, the structure of the parts upon which they are about to operate. More striking instances of the necessity for minute examinations can scarcely be pointed out, than those of the peculiarities in the relations, which are shown by Mr. Swan in the Part now before us to exist between the vagus and sympathetic nerves, in different mammalia. Thus, in the rabbit and hedgehog, which are often the victims of experiments, the prolongation of the sympathetic, which descends from the superior cervical ganglion, passes down the neck to the second cervical, distinctly separated from the trunk of the vagus; but in the pig, according to Mr. Swan, these nerves are slightly connected together laterally, just before the trunk of the sympathetic reaches the second ganglion; but in other instances, among which are the ass and calf, often also the subjects of experiment, the prolongations of the sympathetic and vagus nerves are closely attached to each other, and proceed through the neck as one nerve. The description given by Mr. Swan is as follows:

"In many animals, as the jaguar, dog, fox, ass, calf, and goat, the prolongation through the neck is attached to the trunk of the par vagum, and in the calf has sometimes very small ganglia imbedded in it, which give filaments to accompany small arteries; it leaves the trunk of the par vagum sooner or later at the bottom of the neck to form the inferior cervical ganglion, and become connected with the first thoracic." (p. 113.)

We were particularly interested some years since with this peculiarity in the distribution of the vagus and sympathetic nerves, when examining them in the calf, as admirably illustrating the necessity of carefully examining every structure experimented on, in every instance, before attempting to draw conclusions from the results of our operations; and now, in further confirmation of the necessity for this, we need but refer again to the statements of Mr. Swan, from which it appears that in three varieties of the same species of animal, the common dog, he has found the superior cervical ganglion varying both in size and shape, while in another tribe of animals, the quadrumana, the spheno-palatine ganglion, a part of the sympathetic so distinct in man, is almost or entirely absent. In our own dissection of the pig we have found the prolongation of the sympathetic attached to the trunk of the vagus throughout nearly the whole of its course through the neck, from the first to the second cervical ganglion, just as we have seen it and as it is described by Mr. Swan in the calf. Mr. Swan, however, found it attached to the trunk of the vagus only for a very short distance just before it joins the second ganglion. These variations in the same kind of animal still further show that it is unsafe to draw any conclusion from the results of experiments which are not supported by anatomical evidence derived from actual observation. The description given by Mr. Swan of the connexions of the nerves from the superior cervical ganglion with the branches of the fifth, glosso-pharyngeal, seventh, and ninth, is in perfect accordance with our own examinations of these parts in the pig; and the precise account he has given of the sympathetic and trunk of the vagus, and their relations in the ass, the jaguar, dog, and fox, is so worthy of attention that we cannot forbear quoting it at length.

"In the ass, the sympathetic passes from the trunk of the par vagum nearly as in the calf, but the right inferior cervical ganglion is rather larger, and the left extends to the first thoracic; distinct communications exist between branches from the first thoracic ganglion, the phrenic nerve, and the trunk of the par vagum; the inferior cervical and first thoracic ganglia communicate very much with the recurrent nerves and some branches of each trunk of the par vagum, so as to form a plexus, from which branches proceed to the heart, those of the right side prevailing for the left ventricle, and those of the left for the right; others joined by branches from the trunk of the par vagum and given to the right auricles. In the jaguar, the sympathetic on leaving the trunk of the par vagum passes to the inferior cervical ganglion, which on the right side forms a small ganglion and a still smaller one on the left; these send off branches to encircle the subclavian artery and join the first thoracic ganglion, and give off cardiac branches, which, together with others from the first thoracic ganglion and the trunk of the par vagum on each side, form communications with the recurrent nerves; the branches then pass principally to the ventricles; other branches from the trunk of the par vagum and recurrent, which have communicated with some of those given to the ventricles, pass to the auricles, but some of these are also extended to the ventricles. Branches from the first thoracic ganglion join the par vagum, the recurrent, and phrenic nerves. In the dog and fox the inferior cervical ganglion is intimately connected with the trunk of the par vagum; it is placed at the inner side of the first thoracic, the short prolongation passing from one to the other over and under the subclavian artery, it sends off branches to join others from each trunk of the par vagum and the recurrent for supplying the heart; in the fox it appears almost as if a large branch issued from the first thoracic ganglion to join the trunk of the par vagum, and for many of the cardiac nerves to pass off after this junction. In the dog communications between each inferior cervical ganglion and phrenic nerve can be traced." (pp. 113-4.)

From the above description it will be seen that while experiments may be made on the trunk of the vagus in the rabbit and hedgehog, without injuring the sympathetic, it is scarcely possible to make similar ones in the ass and calf, and still less practicable in the dog and fox, without also including the latter nerve, and consequently complicating the experiments, and rendering the conclusions drawn from them less certain.

Mr. Swan has also noticed other interesting facts connected with the distribution of the sympathetic. Thus he traced branches from the superior mesenteric plexus to several parts of the large mesenteric glands in the jaguar, and found prolongations of the sympathetic from the renal, spermatic, and aortic plexuses forming points of union towards the bottom of the sacrum, but coalescing on the caudal artery, along which the "nerves are continued for supplying the coats of this vessel; in the calf each prolongation terminates on this vessel in the single ganglion, from which a prolongation is continued; in the jaguar each prolongation was traced into the tail, to be ultimately distributed on this artery." (p. 117.) He remarks also on some interesting peculiarities in the human fœtus. Thus in a fœtus of five months the superior cervical ganglion was large, and the conjunction of its branches with the vagus and ninth nerves had a gangliform appearance. In the same fœtus he observed that the spinal marrow was nearly perfect, and reached to the bottom of the sacrum, and that the spinal ganglia were large and well formed; and what is still more interesting, that in a fœtus of four months there was a ganglion at the superior part of the par vagum. This latter fact is particularly worthy of notice, from the circumstance that, as is well known, in the human adult only a very slight enlargement exists on the vagus, just after it has passed out of the cranium, the ganglion which is thus found to exist in the fœtus having almost entirely disappeared. We agree with Mr. Swan that it would be highly interesting to trace the changes that take place in the development of the sympathetic system in the young of different animals. Another valuable fact also noticed by Mr. Swan is that "in the calf......the branches of the sympathetic can be traced close to the anterior and posterior bundles of the spinal nerves with which they coalesce, but cannot be followed to the spinal marrow without violently severing communicating filaments." (p. 120.)

With these valuable facts before us we need scarcely again express our approbation of the anatomical part of Mr. Swan's work, yet we cannot restrain a wish, which we felt strongly when reviewing a former number of this work, that Mr. Swan would be content to confine himself to elucidating the peculiarities of the anatomy of the nervous system, on which his remarks, as being derived from careful and probably often-repeated dissections, have given his statements a degree of authority of which any man might well be proud—instead of attempting to theorize on the uses of the parts, which he is so much prone to do, and in doing which he almost invariably fails. Whenever he departs from the observation of facts his conclusions are almost always hypothetical and erroneous assumptions, totally unworthy of his reputation as a great anatomist. We will select a few instances of these errors from the present Part, in illustration of the justness of our strictures. Take the following:

"Many secretions are offensive to the skin, which has not the protection of mucus, but some things hardly affect it which irritate excessively the intestines. But *it is most probable* that the nearer the nerves of the intestines approach to those of the skin the less uneasy impression any coarse or acrid food will produce, and *consequently* a less speedy evacuation. The *open texture* of ganglia in the herbivorous feeders of mammalia, and in both herbivorous and carnivorous feeders of amphibia, although *it* be easily irritated, may have a longer retention of food than *fleshy* ganglia would have done." (p. 125.)

Again, in pursuing this hypothesis, he says,

"Through the fleshy or thready state of the ganglia of the spinal or sympathetic nerves, it may be presumed that the parts supplied by or communicating with them are made more or less subservient to each other, according to the required condition of every animal. *It is most probable* that the coarser and more irritating the food, the nearer the splanchnic nerves, the semilunar ganglia, and plexuses approach to the thready texture or that of the spinal nerves in mammalia; and this state of the ganglia is found in the animals which have the food transmitted through the intestines the most quickly." (pp. 125-6.)

This favorite hypothesis respecting the thready state of the ganglia he winds up with the following unique addition to his theory.

"As the thready disposition of the ganglia exists both in *the pig* and *boa constrictor*, as well as in the *turtle*, it is not unfavorable to the formation and accumulation of *fat*." (p. 126.)

These are assumptions at which we cannot forbear smiling, and the gratuitous nature of which we are quite certain will fully justify any severity of criticism on our part; but we will give a few more instances of these hypothetical remarks out of the many others with which the present number of Mr. Swan's work abounds.

"*It is probable* that in all animals the more nutritious the food the less it irritates the nerves, and the longer it is in passing through the small intestines; so that even the same articles, if cooked, may have their nutrient parts absorbed in these, but, if raw, will have a much more considerable portion remaining to be extracted in the large; so that the capacity either of the small or large intestines will be very much influenced, not only by the food itself, but its mode of supply, and the animals thus rendered more or less capable *of making particular exertions*." (p. 127.)

Besides this we are gravely told, on the same page, that "the peculiarity of the milk results from the nature of each animal, and is to a certain degree required in the young *on account of the particular state of the sympathetic nerve*." It is indeed much to be lamented that Mr. Swan should be so much inclined to theorize, and while stating so valuable an anatomical fact as that contained in our next quotation, that he should mar the interest attached to it by appending, in the very next sentence, an explanation which we can hardly otherwise designate than as absurd.

"The duodenum, the small intestines, and the cæcum, and a more or less extensive portion of the upper part of the colon, are furnished by the superior mesenteric plexus, which has a considerable admixture of branches from the par vagum; in man, the baboon, jaguar, dog, fox, calf, ass, and pig, the remaining portion of the large intestines, as far as the rectum, is supplied by the sympathetic. In man and the baboon the descending portion of the colon has an extraordinary quantity of nerves from the aortic and hypogastric plexuses, *in all probability* both for endowing it with higher powers for *preserving it from the acrimony of, and for expediting the expulsion of, the feculent matter*." (p. 131.)

These assumptions, in proof of which we have not a single pathological or physiological fact, appear to have been printed merely because they happened to be in the imaginings of the author, since we cannot discover that he has any general views or principles to which they may be subsidiary. Such inconsiderate intrusions of mere surmises like these into anatomical works have done much harm to the progress of science; and have tended to render our knowledge unstable. While noticing the defects of this work, and before again turning to those parts of it on which we can offer nothing but praise, we may briefly remark that we could have wished the descriptions and observations had been written with a little more attention to literary accuracy; since in many sentences the style is so confused and incorrect that we are not quite sure that we have always rightly understood the meaning of the author.

In returning to the more agreeable part of our duty, that of pointing out what appears more especially deserving of notice, we are particularly interested with the description of the connexion that is shown to exist between the nerves of the uterus and mammæ, in the ass. Mr. Swan says that

"From the third lumbar ganglion of the sympathetic a large branch passes to the aortic plexus, and from this the internal spermatic nerve is given off and distributed to the Fallopian tubes, the ovary, and uterus, and the round ligaments. From the same part of the aortic plexus the hypogastric is given off, which in passing down sends filaments to the uterus and the remains of the umbilical artery, and its surrounding membranæ; *it is most probable* that similar nerves accompany these arteries and the umbilical cord in the fœtus; it then forms an intimate plexus, with branches from the fourth, fifth, and sixth sacral nerves, to be distributed on the bladder, vagina, and rectum. The third lumbar ganglion of the sympathetic also sends a large branch to join the external spermatic nerve, a great part of which is distributed on the mamma. The close connexion between this organ and the uterus, with its appendages, is thus clearly demonstrated; and although it cannot be shown in the human body or in many animals, it is in the highest degree probable that a similar participation of function is effected through the sympathetic nerve." (p. 132.)

The ascertainment of this connexion between the nerves of the uterus and mammæ, even in one animal, is an interesting fact. He has been unable, however, to demonstrate a distinct connexion between the mammary and uterine nerves from the sympathetic system in some other quadrupeds, as in the sow, in which the mammæ are numerous, and distributed along the whole of the pectoral and abdominal surface of the body. In that animal he found the nerves that supply the mammæ derived from the intercostal and other spinal nerves, and hence he concludes that the mammæ are supplied with branches from the sympathetic system through means of the branches that accompany the arteries, and also those which are bound up in the sheaths of the spinal nerves, which, as is well known, are directly connected with the sympathetic system at their origin.

There is another anatomical fact pointed out by Mr. Swan which we regard as of equal interest with that we have just noticed; it is the continuation of the posterior branches of the vagus nerve into, and their becoming united with, the great mesenteric plexus, as shown on the same plate, (xxvii., fig. 1, 9,) on which the connexion of the mammary and uterine nerves is demonstrated. This is in accordance with what has been ascertained by Weber and Müller of the distribution of this nerve in some of the lower vertebrata: the first of whom found the vagus distributed to a great part of the alimentary canal in the snakes, while the latter states that in the myxinoid fishes the vagus extends as far as the anus; and we doubt not that such would be found to be the case in the higher vertebrata, if the connexions which Mr. Swan has shown it to form with the mesenteric plexus could be traced through the intricate combination of its fibres to their termination.

The remaining portion of the present part is devoted to some general descriptions of the cerebral and respiratory nerves, and of the structure of the brain and spinal cord, in mammalia; but on these subjects we have not remarked anything which has appeared to us particularly worthy of notice. The extracts we have given will sufficiently indicate our opinion of the value of this work, which, indeed, as an anatomical one can scarcely be too much commended.

